# The relation between physical activity and cardiac autonomic dysfunction in patients with chronic obstructive pulmonary disease

**DOI:** 10.1371/journal.pone.0353482

**Published:** 2026-08-25

**Authors:** Fien Hermans, Eva Arents, Astrid Blondeel, Wim Janssens, Nina Cardinaels, Patrick Calders, Thierry Troosters, Eric Derom, Heleen Demeyer

**Affiliations:** 1 Department of Rehabilitation Sciences, Ghent University, Ghent, Belgium; 2 Department of Rehabilitation Sciences, KU Leuven, Leuven, Belgium; 3 Department of Chronic Diseases, Metabolism and Aging (CHROMETA) - BREATHE, KU Leuven, Leuven, Belgium; 4 Clinical department of Respiratory Diseases, University Hospitals Leuven, Leuven, Belgium; 5 Department of Respiratory Medicine, Ghent University Hospital, Ghent, Belgium; Kasetsart University Faculty of Veterinary Medicine Kamphaengsaen Campus, THAILAND

## Abstract

**Background:**

Cardiac autonomic dysfunction is common in patients with chronic obstructive pulmonary disease (COPD). Although exercise training improves cardiac autonomic function, the effects of changing physical activity on cardiac autonomic function remain unknown.

**Objective:**

This study aimed to (1) investigate the association between cardiac autonomic function, exercise capacity and physical activity cross-sectionally and (2) relate changes in physical activity, as a result of a 6-month coaching program, to changes in cardiac autonomic function.

**Methods:**

Autonomic function was assessed objectively using the Polar H10 sensor, measuring (1) resting heart rate during sleep; (2) heart rate variability (SDNN, RMSSD, LF/HF ratio and SD2/SD1 ratio) during sleep and (3) heart rate recovery following the 6-minute walking test (6MWT), and subjectively using the COMPASS 31 questionnaire at baseline and 6-months follow-up. Physical activity (steps per day and movement intensity during walking) was measured using accelerometery. Cross-sectional and longitudinal associations were evaluated.

**Results:**

In 36 patients with COPD (69% male, mean ± SD age 69 ± 7 years, FEV_1_ 53 ± 13%pred, 28% taking β-blockers), baseline cross-sectional analyses showed weak to moderate associations between heart rate recovery and movement intensity during walking (r_s_ = 0.45) and distance covered during 6MWT (r_s_ = 0.55). Longitudinal analyses over 6-months follow-up showed that changes in daily step count were inversely associated with changes in resting heart rate (r_s_ = −0.48), LF/HF ratio (r_s_ = −0.35) and SD2/SD1 ratio (r_s_ = −0.28) and that changes in movement intensity during walking were inversely associated with changes in SD2/SD1 ratio (r_s_ = −0.51) in patients not taking β-blockers.

**Conclusion:**

Weak cross-sectional associations were observed between certain parameters of cardiac autonomic function, exercise capacity and physical activity. Increasing the amount of physical activity showed a weak relation with lowering resting heart rate over 6 months in patients not taking influencing medication.

## Introduction

The autonomic nervous system is responsible for maintaining the body’s homeostasis through a complex and dynamic interaction of sympathetic and parasympathetic branches [1]. This system regulates various vital functions, including heart rate, blood pressure, respiratory rate and body temperature, in response to internal and external stimuli [1]. An imbalance in the autonomic nervous system occurs in various chronic disorders such as cardiovascular disease, Parkinson’s disease, diabetes mellitus and also chronic obstructive pulmonary disease (COPD) [2–6].

Compared to healthy individuals, patients with COPD have been characterised with an increased resting heart rate, a decreased heart rate variability, a lower heart rate recovery following exercise and more self-reported autonomic symptoms reflecting a predominance of sympathetic activity [5,7–10]. These alterations in autonomic function have been attributed to several factors, including hypoxemia, bronchodilator use, and oxidative stress [5,11] and have been related to disease severity and may predict the occurrence of severe acute exacerbations [7,9,12]. Moreover, impaired autonomic dysfunction negatively impacts both quality of life and mortality [13,14]. Nevertheless, autonomic function is barely evaluated in clinical routine despite the development of user friendly equipment (e.g., Polar H10 chest band), which enables these parameters of autonomic function to be assessed non-invasively and for extended periods of time [15]. In this, measuring heart rate variability plays an important role in the assessment of autonomic function in general as it has been related to gastrointestinal symptoms, constipation, diarrhea and excessive sweating, all regulated by the autonomic nervous system [16–18].

Exercise intolerance is a common but treatable trait in patients with COPD [19]. Various studies in patients with COPD indicate that cardiac autonomic function is associated with functional exercise capacity, as higher resting heart rate and delayed heart rate recovery are linked to reduced exercise capacity [20–22]. This association is confirmed by observations showing that 4–12 weeks of exercise training, a cornerstone of treatment in patients with COPD [19], not only improves dyspnoea, exercise intolerance and quality of life, but also key measures of autonomic function, including resting heart rate, heart rate variability and heart rate recovery [23–25].

Patients with COPD show lower levels of physical activity compared to healthy peers. Both reduced amount and intensity of physical activity are associated with an increased risk of developing acute exacerbations and mortality [26,27]. Previous cross-sectional studies indicated that parameters of cardiac autonomic function (i.e., heart rate variability and heart rate recovery) are positively correlated with volume of physical activity in patients with COPD [28,29]. Various coaching interventions aimed at enhancing physical activity have been investigated in this population [30]. Nonetheless, it remains unexplored whether these physical activity coaching interventions, which typically require durations longer than 3 months to achieve meaningful and sustained behavioural change, can positively impact cardiac autonomic function.

The aims of the present study are to investigate (1) the cross-sectional associations between physical activity, functional exercise capacity and parameters of cardiac autonomic function at baseline and (2) the relationship between changes in physical activity with changes in cardiac autonomic function in patients with COPD over a 6-month period. We hypothesize that there is an association between both the amount and intensity of physical activity as well as functional exercise capacity and parameters of cardiac autonomic function and that a physical activity coaching intervention would not only increase the amount and intensity of physical activity after 6 months but also alter parameters of cardiac autonomic function eventually resulting in improvements in resting heart rate, heart rate variability and heart rate recovery.

## Methods

### Study population and design

This study was nested in a larger randomised controlled trial investigating the long-term effects of a physical activity coaching intervention in patients with COPD (NCT04139200). For the present sub-study, patients were recruited at Ghent University Hospital (Ghent, Belgium) between September 30, 2021 and April 12, 2023. Patients were eligible for participation if the following criteria were met: (1) age over 40 years; (2) a smoking history of at least 10 pack years; (3) a clinical diagnosis of COPD (confirmed by spirometry (FEV_1_/FVC < 0.70)) and (4) no history of exacerbations in the last month before inclusion. Patients actively involved in a multidisciplinary rehabilitation program, on the waiting list for a lung transplantation, having musculoskeletal comorbidities preventing to improve physical activity or being unable to learn to work with a smartphone were excluded.

For the present sub-study, data were obtained at baseline and after 6-months follow-up. The study was approved by the ethical committee of Ghent University Hospital (BC-10267) and all patients provided written informed consent prior to data collection.

### Data collection

#### Patients characteristics.

Following data were collected at baseline: (1) sociodemographic data (age, sex, height and weight); (2) pharmacological treatment; (3) exacerbation history in the last 12 months before inclusion; (4) smoking history; (5) post-bronchodilator spirometry following the American Thoracic Society (ATS)/European Respiratory Society (ERS) guidelines; (6) dyspnoea using the modified Medical Research Council scale (mMRC); (7) health status using the COPD Assessment Test (CAT) and (8) isometric quadriceps force with the patient in a seated position with 90° hip and knee flexion as the best of three attempts [31].

#### Autonomic function.

Cardiac autonomic function was assessed at baseline and 6-months follow-up using the Polar H10 sensor chest band (Polar Electro Oy, Kempele, Finland), a validated device for recording heart rate and heart rate variability, linked to the corresponding Polar Ignite 2 watch, which served as external data logger, ensuring continuous data collection for up to 100 hours [15]. Patients were instructed to wear both devices for 4 consecutive nights and maintain a diary including intake of medication, time of waking up and time of going to bed. The Polar H10 sensor recorded data at 1-second time intervals, and all data were extracted using the Polar Flow web service (see supplementary materials). The following parameters were measured:

***Resting heart rate:*** Resting heart rate (RHR) was defined as the average heart rate during the night (sleep time as reported in the diary).

***Heart rate variability:*** Heart rate variability (HRV) was measured during the night (sleep time as reported in the diary). The beat-to-beat RR intervals were transferred to Kubios HRV Scientific Software (version 4.0.3, Kuopio, Finland) to analyse HRV [32]. HRV analyses included both linear and non-linear measurements, based upon the guidelines of the European Society of Cardiology and the North American Society of Pacing and Electrophysiology [33]. Linear measurements in the time domain analyses included the standard deviation of RR intervals (SDNN), expressed in milliseconds and the root mean square of successive RR interval differences (RMSSD), expressed in milliseconds. Linear measurements in the frequency domain analyses were evaluated by fast Fourier transform algorithms to analyse the low frequency/high frequency ratio (LF/HF ratio). Non-linear measurements were analysed using Poincaré plots (SD2/SD1 ratio).

***Heart rate recovery:*** Heart rate recovery (HRR) was evaluated following the 6-minute walking test (6MWT), performed according to the ATS/ERS guidelines [34]. Heart rate was continuously measured during the 6MWT. After completion of the 6MWT, patients were asked to rest on a portable chair. HRR was defined as the difference between the heart rate at the end of the 6MWT and the heart rate after 1 minute of rest.

***COMPASS 31 questionnaire:*** Autonomic nervous system symptoms were subjectively assessed using the composite autonomic symptom score (COMPASS 31). This questionnaire evaluates the severity and distribution of symptoms as well as the autonomic function capacity across six domains: orthostatic intolerance, vasomotor, secretomotor, gastrointestinal, bladder and pupillomotor function and renders a total score ranging from 0 (optimal) to 100 (poor) [35].

#### Physical activity and functional exercise capacity.

Physical activity was objectively measured using the Dynaport MoveMonitor (McRoberts, The Hague, The Netherlands) at baseline and 6-months follow-up. Patients were instructed to wear this validated tri-axial accelerometer for 7 consecutive days during waking hours [36]. According to recommendations, a valid measurement was defined as a minimum of two weekdays with each day at least 8 hours of wearing time [37]. Weekend days were excluded for the analyses. Day-by-day data were exported using the McRoberts software (version 2.0.1)) to retrieve adaily step count (n/day) and movement intensity (MI) during walking (m/s^2^). Functional exercise capacity was measured at baseline and 6-months follow-up as the greatest distance covered out of two 6MWT, performed according to the ATS/ERS guidelines [34] and related to reference values [38].

#### Coaching intervention.

Patients received a full or light physical activity coaching intervention during the follow-up period, according to randomisation. Both groups were pooled in the present sub-study. Details of this coaching intervention have been published previously [39]. In summary, the intervention contained the following components for both groups: (1) a one-to-one interview with the coach discussing motivation, self-efficacy and barriers; (2) a wrist-worn consumer-based wearable (Fitbit Charge 4, Fitbit Inc., San Francisco, CA) worn on a daily basis and providing direct feedback (expressed as daily step count); (3) a smartphone application (m-PAC, AppsOnly, Leuven, Belgium) providing patients an overview of their daily activities. Patients in the full coaching group received an individualised adaptive step goal with daily and weekly feedback and instructions to let their step goal progress over time. Patients in the light coaching group received a general fixed step goal based on their exercise capacity; (4) contact with the coach was initiated by the coach in the full coaching group in a priori defined situations (e.g., failed to improve their physical activity level, having technical issues and reporting a change in medication).

### Statistical analysis

All statistical analyses were performed using the SAS statistical package (version 9.4, SAS Institute, Cary, North Carolina, USA). Data are presented as mean ± standard deviation (SD) or median (Q1-Q3), as appropriate after testing for normality using Shapiro-Wilk test and inspecting QQ-plots. Statistical significance was set at p < 0.05 for all analyses. Analyses were performed on complete cases, with no imputation techniques applied for missing data.

Cross-sectional associations at baseline between parameters of cardiac autonomic function, functional exercise capacity and physical activity were evaluated via Spearman correlation. Correlation was interpreted using the following cut-offs: r_s_ < 0.30 classified as ‘no correlation’; r_s_ = 0.30–0.50 as ‘weak correlation’; r_s_ = 0.50–0.70 as ‘moderate correlation’; r_s_ = 0.70–0.90 as ‘strong correlation’ and r_s_ > 0.90 as ‘very strong correlation’ [40].

Longitudinal changes in physical activity, functional exercise capacity and parameters of cardiac autonomic function between baseline and 6-months follow-up were assessed via Wilcoxon signed rank test. A Spearman correlation was performed to investigate the association between changes in physical activity and changes in parameters of cardiac autonomic function over the 6-month follow-up period.

The analyses were repeated excluding patients on stable doses of β-blockers because, due to the impact of this medication on heart rate and HRV [41], we expect this to be a modifier of the effect.

Finally, two sensitivity analyses were conducted. First, analyses were conducted with autonomic function measured during the clinical visit using the Polar H10 sensor chest band, during a 10-minute rest period in a quiet room with the patient in a supine position. RHR and HRV were defined as the average heart rate and HRV after 5 minutes of rest within this 10-minute rest period, respectively [42]. This sensitivity analysis was a priori planned due to foreseen missing data caused by technical issues during the measurements conducted over 4 consecutive nights. Second, the analyses were repeated excluding patients diagnosed with and treated for obstructive sleep apnoea syndrome, due to the impact of sleep apnoea on cardiac autonomic function [43].

## Results

### Patient characteristics

A total of 36 patients with COPD were included in the present study. Fifty-six percent of the included population had mild to moderate COPD. Patients had a relatively well preserved quadriceps force (78 ± 20%pred) and functional exercise capacity (77 ± 12%pred). 10 out of 36 patients (28%) were taking β-blocking agents for the indication of hypertension (Table 1), 7 patients (19%) were diagnosed with sleep apnoea.

**Table 1:** Baseline characteristics of patients included in the analyses, expressed as mean ± SD, median (Q1-Q3) or proportions.

Variable	Patients with COPD (n = 36)
**Age (years)**	69 ± 7
**Male, n (%)**	25 (69)
**BMI (kg/m**^**2**^)	27 ± 6
**FEV**_**1**_ **(% pred)**	53 ± 13
**GOLD stage**	
**I-II, n (%)**	20 (56)
**III-IV, n (%)**	16 (44)
**Frequent exacerbators, n (%)**	4 (11)
**Current smokers, n (%)**	3 (8)
**6MWD (m)**	477 ± 88
**6MWD (%pred)**	77 ± 12
**Quadriceps force (%pred)**	78 ± 20
**COMPASS 31 (score, 0–100)**	19 ± 12
**mMRC (score, 0–4)**	2 (1–3)
**CAT (score, 0–40)**	17 ± 5
**Mean steps per day (n/day)**	5116 ± 2542
**Movement intensity during walking (m/s²)**	1.7 ± 0.2

6MWD = Six-minute walking distance; BMI = Body mass index; CAT = COPD assessment test, a higher score indicating a worser health status; COMPASS 31 questionnaire = composite autonomic symptom score, a higher score indicating more symptoms of autonomic dysfunction; FEV_1_ = Forced expiratory volume in the first second; GOLD = Global initiative for chronic obstructive lung disease; mMRC = modified medical research council dyspnoea scale, a higher score indicating more dyspnoea.

**Fig 1:**
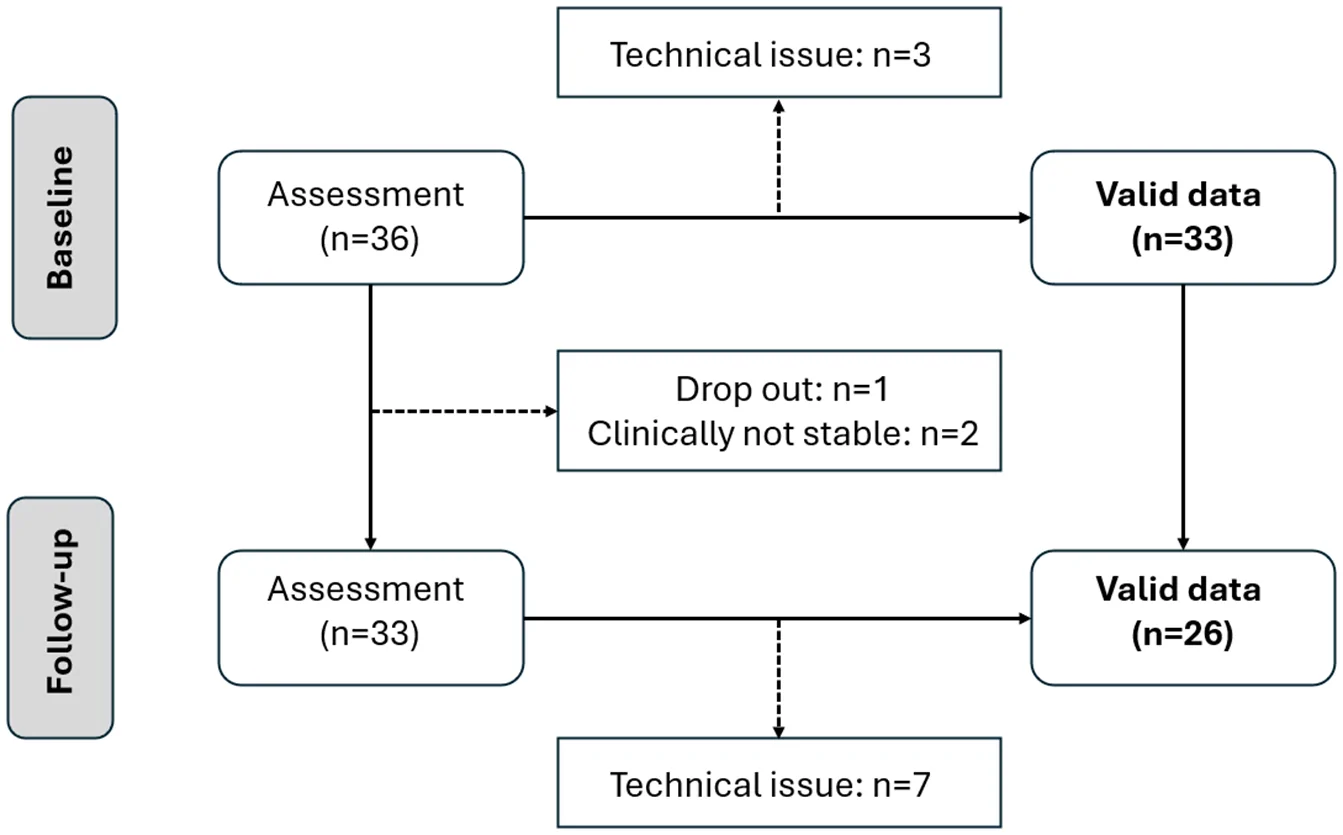
Flowchart of patients included in the study. Due to technical issues during the assessment over 4 consecutive nights, valid data is available for 33 patients at baseline and 26 patients at 6-months follow-up.

Thirty-three patients were followed-up (Fig 1). Technical issues during the assessment over 4 consecutive nights occurred in 3 patients at baseline and 7 patients at 6-months follow-up. Baseline characteristics of patients included in the 24 hour assessment are shown in S1 Table.

Baseline characteristics of patients who completed the study (n = 26) and those who did not (n = 10) are presented in S2 Table. Both groups did not differ in parameters of autonomic function or physical activity at baseline, whereas non-completers had significantly lower lung function and reduced functional exercise capacity compared to completers.

### Cross-sectional associations at baseline

#### Cardiac autonomic function.

**Fig 2:**
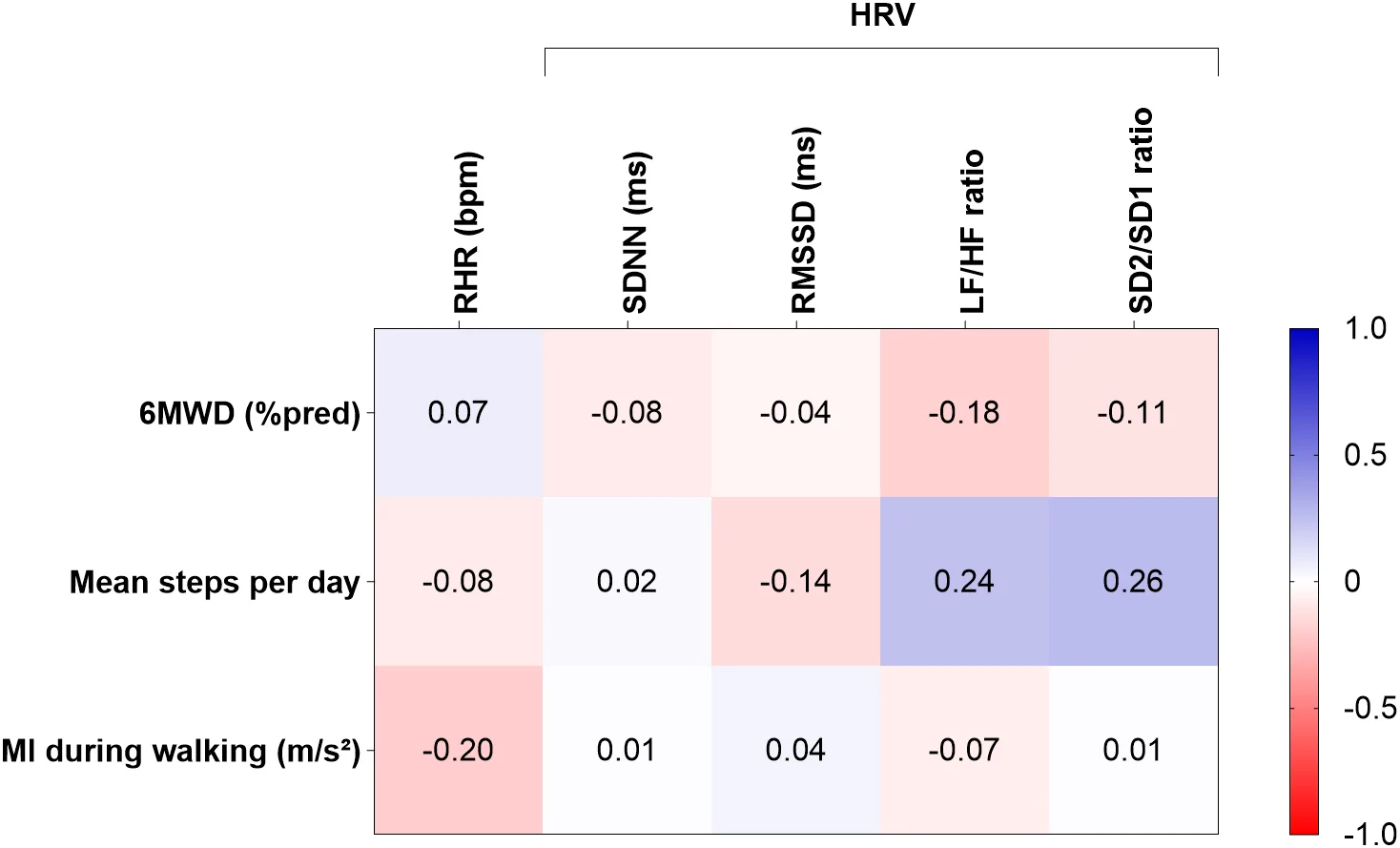
Spearman correlations between autonomic function (24 hour assessment) and functional exercise capacity or physical activity in patients with COPD at baseline. Red = negative correlation and blue = positive correlation, with a darker colour representing a stronger correlation. 6MWD = Six-minute walking distance; bpm = Beats per minute; HRV = Heart rate variability; LF/HF = Low frequency/High frequency; MI = Movement intensity; ms = Milliseconds; RHR = Resting heart rate; RMSSD = Root mean square of successive RR interval differences; SD1 = Short-term variability (standard deviation perpendicular to the line of identity); SD2 = Long-term variability (standard deviation along the line of identity); SDNN = Standard deviation of RR intervals.

Cardiac autonomic function was not associated with functional exercise capacity, mean steps per day or MI during walking at baseline, see Fig 2. Excluding patients taking β-blockers did not affect the strength of the associations (S1 Fig).

#### Heart rate recovery and symptom experience.

**Fig 3:**
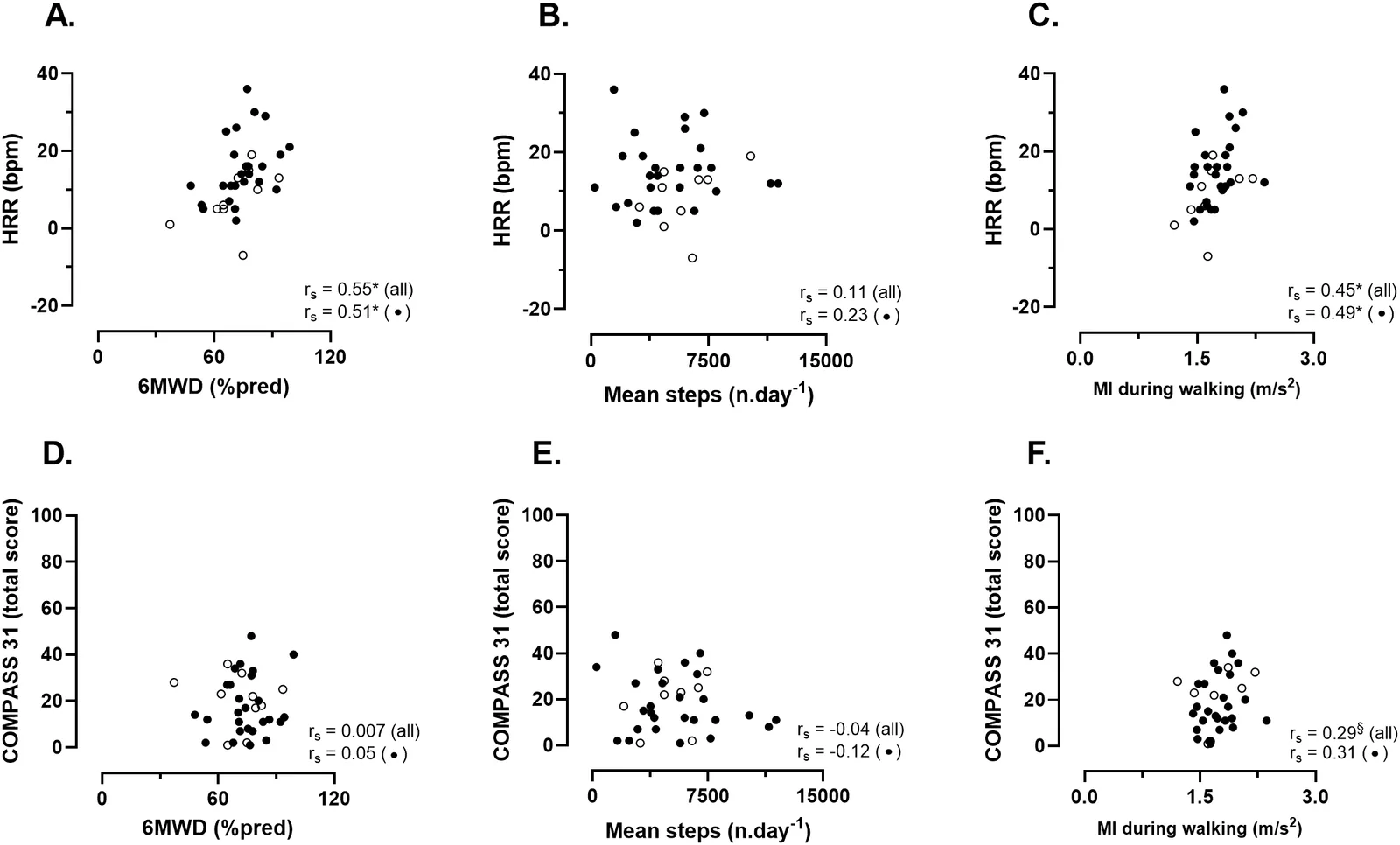
Scatter plots of the associations between heart rate recovery (HRR) following the 6MWT and (A) 6-minute walking distance (expressed as % predicted), (B) daily step count and (C) movement intensity during walking and between total score of COMPASS 31 questionnaire and (D) 6-minute walking distance (expressed as % predicted), (E) daily step count and (F) movement intensity during walking. Patients taking β-blockers are depicted in the open dots (N = 10). 6MWD = Six-minute walking distance; bpm = Beats per minute; HRR = Heart rate recovery; MI = Movement intensity; * = Statistically significant (p-value < 0.05); ^§^ = Trend towards significance (p-value < 0.10).

In these cross-sectional analyses (n = 36), HRR was moderately associated to functional exercise capacity and weakly to MI during walking (r_s_ = 0.55 and r_s_ = 0.45, respectively) (Fig 3). Excluding patients taking β-blockers did not alter the results (r_s_ = 0.51 and r_s_ = 0.49, respectively). No association between COMPASS 31 questionnaire (total score) and functional exercise capacity or physical activity was found at baseline (Fig 3).

### Longitudinal associations

Physical activity coaching did not significantly change mean steps per day (median (Q1-Q3) ∆ 525 (−632–1324) steps/day), MI during walking (∆ 0.00 (−0.09–0.09) m/s^2^) or distance covered during 6MWT (∆ 4 (−17–21) m) (p ≥ 0.10 for all) over 6 months. Likewise, parameters of cardiac autonomic function did not change between baseline and 6-months follow-up, except of the domain of the COMPASS 31 questionnaire assessing bladder function (p = 0.037) (Table 2). Results were similar after excluding patients taking β-blockers from the analyses (S3 Table).

**Table 2:** Changes (median (Q1-Q3)) in parameters of autonomic function in patients with COPD between baseline and 6-months follow-up (6M).

Parameter	Baseline Median (Q1-Q3)	6M Median (Q1-Q3)	∆ Median (Q1-Q3)	p-value
Resting heart rate				
No β-blocker use (bpm)	69 (64–76)	68 (67–72)	0 (−3–3)	0.99
β-blocker use (bpm)	62 (60–66)	64 (63–66)	2 (−3–4)	0.64
Heart rate variability				
SDNN (ms)	25 (14–38)	24 (20–34)	0 (−6–6)	0.99
RMSSD (ms)	14 (12–36)	24 (14–32)	1 (−5–6)	0.52
LF/HF ratio	2 (1–6)	3 (1–6)	0 (−1–1)	0.81
SD2/SD1 ratio	2 (2–3)	2 (1–3)	0 (0–0)	0.60
Heart rate recovery (bpm)	13 (6–16)	16 (8–21)	2 (−2–6)	0.20
COMPASS 31				
Total score	17 (11–27)	17 (7–23)	−1 (−6–2)	0.13
Orthostatic function	0 (0–16)	0 (0–12)	0 (0–0)	0.99
Vasomotor function	0 (0–0)	0 (0–0)	0 (0–0)	0.64
Secretomotor function	4 (0–6)	2 (0–6)	0 (−2–0)	0.43
Gastrointestinal function	4 (1–7)	2 (1–5)	0 (−2–0)	0.07^§^
Bladder function	1 (0–3)	0 (0–2)	0 (−1–0)	0.04*
Pupillomotor function	1 (1–2)	1 (1–2)	0 (0–1)	0.75

Bpm = Beats per minute; COMPASS 31 questionnaire (0–100), a higher score indicating more symptoms of autonomic dysfunction; Orthostatic function (0–40); Vasomotor function (0–5); Secretomotor function (0–15); Gastrointestinal function (0–25); Bladder function (0–10) and Pupillomotor function (0–5); LF/HF = Low frequency/High frequency; ms = Milliseconds; RMSSD = Root mean square of successive RR interval differences; SD1 = Short-term variability (standard deviation perpendicular to the line of identity); SD2 = Long-term variability (standard deviation along the line of identity); SDNN = Standard deviation of RR intervals. * = Statistically significant (p-value < 0.05); ^§^ = Trend towards significance (p-value < 0.10).

#### Cardiac autonomic function.

**Fig 4:**
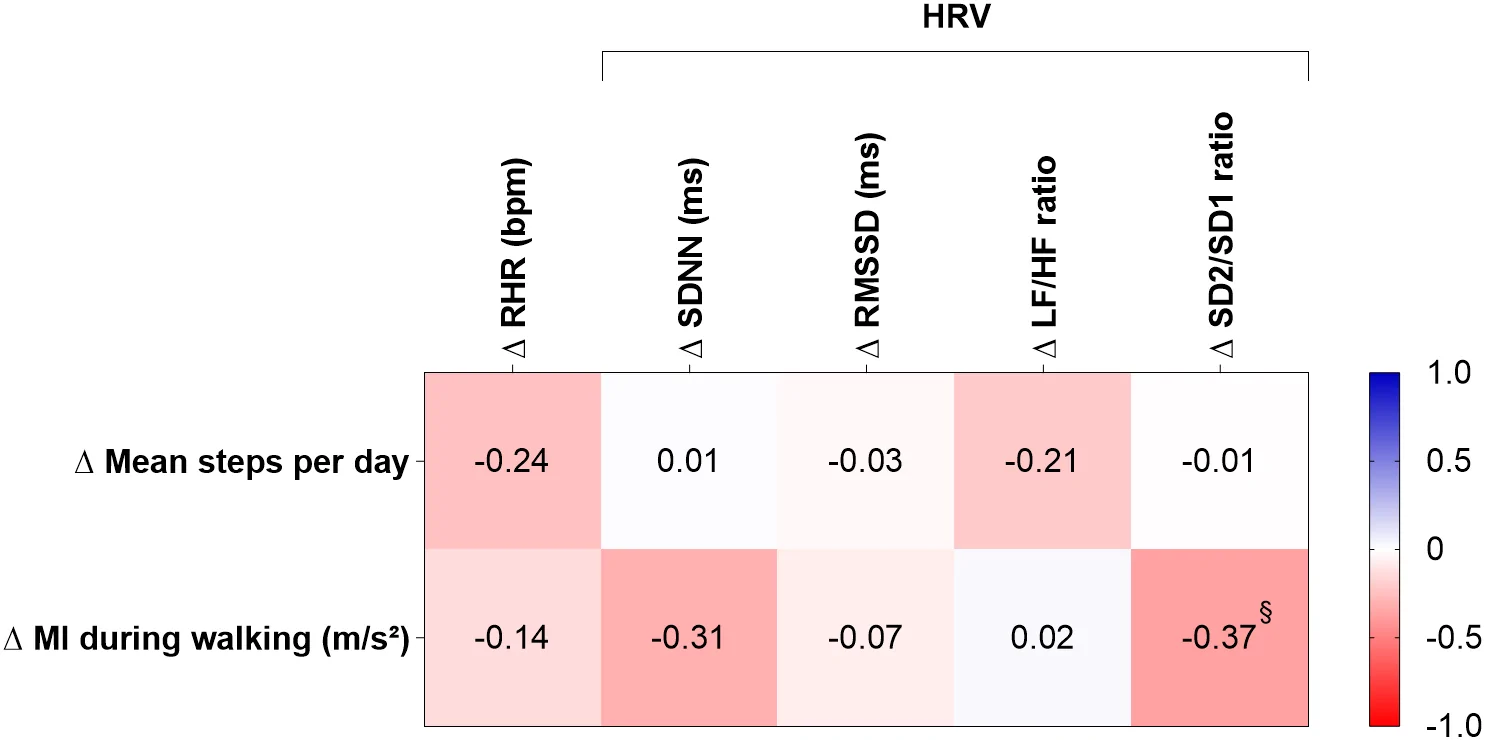
Spearman correlations between changes in physical activity and changes in autonomic function (24 hour assessment) in patients with COPD between baseline and 6-months follow-up. Red = negative correlation and blue = positive correlation, with a darker colour representing a stronger correlation. bpm = Beats per minute; HRV = Heart rate variability; LF/HF = Low frequency/High frequency; MI = Movement intensity; ms = Milliseconds; RHR = Resting heart rate; RMSSD = Root mean square of successive RR interval differences; SD1 = Short-term variability (standard deviation perpendicular to the line of identity); SD2 = Long-term variability (standard deviation along the line of identity); SDNN = Standard deviation of RR intervals. ^§^ = Trend towards significance (p-value < 0.10).

Changes in physical activity were not associated with changes in RHR (Fig 4). When excluding patients on stable doses of β-blockers, the change in amount of physical activity was negatively related to the change in RHR, change in LF/HF ratio and change in SD2/SD1 ratio (r_s_ = −0.48, r_s_ = −0.35 and r_s_ = −0.28, respectively) (S2 Fig). The change in MI during walking was weakly associated with the change in SDNN and change in SD2/SD1 ratio (r_s_ = −0.31 and r_s_ = −0.37, respectively), while a moderate association between change in MI and change in SD2/SD1 ratio was observed when excluding patients on stable doses of β-blockers (r_s_ = −0.51), see Figs 4 and S2.

#### Heart rate recovery and symptom experience.

Changes in physical activity were not related to changes in HRR (r_s_ = −0.06 (mean steps per day) and r_s_ = −0.29 (MI during walking)). The change in amount of physical activity was weakly and inversely related to the change in gastrointenstinal function, measured by the COMPASS 31 questionnaire (r_s_ = −0.41), while a moderate inverse association was observed when excluding patients taking β-blockers (r_s_ = −0.56).

### Sensitivity analyses

Patients in whom autonomic function was assessed during the clinical visit (n = 35) were included in the first sensitivity analyses (baseline characteristics in S4 Table). Cross-sectional and longitudinal associations were closely align with the associations of the 24 hour assessment (S3 and S4 Figs). Second, in line, exclusion of patients with sleep apnoea (n = 7) did show comparable cross-sectional and longitudinal associations.

## Discussion

The present study, which investigated the associations between parameters of cardiac autonomic function, functional exercise capacity and physical activity, showed that HRV is related to the amount of physical activity, whereas RHR and HRR following the 6MWT are both related to functional exercise capacity and the intensity of physical activity measured during daily life. An increase in the intensity of physical activity during 6 months is associated with a decrease in SDNN and SD2/SD1 ratio. In addition, only in patients not taking β-blockers, an increase in the amount of physical activity after 6 months is associated with a decrease in RHR, LF/HF ratio and SD2/SD1 ratio.

Cardiac autonomic function, particularly RHR and HRV, has received considerable attention in recent years due to its association with mortality in both healthy individuals and patients with COPD [9,44,45]. Moreover, as in healthy individuals, RHR measured during a clinical visit has been inversely related to exercise capacity in patients with COPD [20,46]. Our sensitivity analysis based on measures during the clinical visit confirmed this negative, but weak association between RHR and exercise capacity. However, we were unable to confirm the findings of previous studies showing that patients with COPD who were more physically active (self-reported or objectively measured) had a lower RHR compared to those who were inactive [20,47]. The small sample size of our study and differences in methodology might explain this discrepancy. In contrast to most previously published studies, in which RHR is assessed at rest during a 5–10 minutes interval, we measured RHR during sleep in accordance with recent recommendations [48]. Although previous research in the general population observed a strong correlation between these two methods, the RHR measured during a 10-minute rest period in a supine position was higher than RHR measured during sleep [46], a finding confirmed by the present study (71 bpm versus 69 bpm, p = 0.07).

Studies investigating the association between HRV and exercise capacity in patients with COPD are scarce, with existing literature suggesting no association between both parameters [20,28]. This is consistent with the results from the present study. In terms of physical activity, literature is inconsistent [20,28]. An unexpected finding, from the 5 minute assessment, was that the amount of physical activity was weakly related to HRV (expressed as LF/HF ratio and SD2/SD1 ratio), in contrast to our initial hypothesis.

To the best of our knowledge, this is the first study investigating the longitudinal associations between changes in physical activity and changes in cardiac autonomic function. However, the result of the physical activity coaching (+525 steps/day) was much lower than expected [49] and not statistically significant in the present cohort, potentially blunting the tested associations. In patients with COPD not taking β-blockers, changes in physical activity levels were inversely related to changes in RHR, as expected. This is consistent with observations in patients with chronic diseases, where an increase in objectively measured physical activity has been associated with a lower RHR [50]. However, the association was attenuated when all patients were included in the analyses, likely due to the pharmacological impact of β-blockers on cardiac regulation [51]. Moreover, our analyses showed that a change in MI during walking was inversely related to a change in SD2/SD1 ratio, reflecting the sympathovagal balance. A reduced SD2/SD1 ratio, primarily due to an increase in SD1 rather than a decrease in SD2 in the present study, implies an increased vagal tone and reduced sympathetic activity in patients engaging in more intense walking in daily life [52]. Additionally, an increase in daily steps was associated with a decrease in LF/HF ratio and SD2/SD1 ratio when excluding patients on β-blockers. These decreases indicate an improved autonomic balance characterised by increased parasympathetic activity and decreased sympathetic activity. The improvements in vagal tone related to increased physical activity were more pronounced in non-linear measurements of HRV, which are often more sensitive compared to linear measurements (time domain and frequency domain analyses) [53].

To minimise the potential impact of medication changes on autonomic function, all patients were included during a stable situation while receiving their regular maintenance therapy, which included inhaled bronchodilators in all participants. Given the uniformity of this treatment across the cohort, it is unlikely to have introduced bias into our findings. In addition, we implemented a proactive monitoring strategy to detect acute exacerbations of COPD. When such changes occurred, follow-up measurements were postponed until the participant had returned to a stable condition, defined as at least four weeks after completing treatment.

A systematic review concluded that exercise training programs incorporating vigorous aerobic exercises hold promise for enhancing cardiac autonomic function (HRV (time domain analyses) and HRR) in patients with COPD. Both SDNN and RMSSD improved after performing such exercises, with average increases ranging between 5–10 milliseconds and 3–13 milliseconds, respectively [24]. Overall, increases in SDNN and RMSSD in the present study were smaller than observed in earlier studies and not statistically significant, even in patients who increased their step count by 1000 steps per day over 6 months, identified as the minimally clinically important [49] (1 (−6–8) milliseconds, p = 0.74 and −1 (−7–8) milliseconds, p = 0.95, respectively)). This suggests that beyond merely emphasizing the amount of physical activity, engaging in higher intensity exercises is probably crucial for improving various parameters of cardiac autonomic function [24]. Previous research in physically inactive adults also supports this, showing that high-intensity interval training offers additional benefits compared to continuous moderate training concerning HRV [54]. The association between changes in exercise capacity and cardiac autonomic function could not be tested in the present study because, as expected, physical activity coaching did not change the exercise capacity in our patients.

Our findings confirm the previously established delayed parasympathetic response following a submaximal exercise test (HRR after 6MWT (mean ± SD): 14 ± 9 bpm) in patients with COPD [12,29]. Prior studies demonstrated that HRR is weakly associated with the distance covered during the 6MWT, being consistent with our findings [21,22]. Importantly, the magnitude of the decline in heart rate after the submaximal test is moderately related to the increase of the heart rate during the 6MWT.

Studies investigating the relationship between the autonomic symptoms, measured by the COMPASS 31 questionnaire, and exercise capacity or physical activity in patients with COPD have not been published so far, precluding comparison of our findings.

Our results support the measurement of parameters of cardiac autonomic function in patients with COPD (both subjectively and objectively) in daily practice. The observed positive association between increased physical activity and improved autonomic function, reflected in lower resting heart rate and improved sympathovagal balance, suggests an additional health benefit of physical activity promotion in patients with COPD. However, the impact of our intervention on physical activity is limited, possibly blunting the results. Therefore, further exploration into interventions that more effectively enhance physical activity and their impact on cardiac autonomic function parameters is warranted. While the associations observed in the present study were mostly weak to moderate, they are still of clinical and scientific relevance. The observed associations, especially when considering objective and longitudinal measures, contribute novel insights to the field.

### Strengths and limitations

Our results provide an interesting insight in the cross-sectional and longitudinal associations between cardiac autonomic function, exercise capacity and objectively measured physical activity in patients with COPD. We determined parameters of cardiac autonomic function (i.e., RHR and HRV) based on highly detailed 24 hour data measured with the Polar H10 sensor, according to recommendations, complemented by the COMPASS 31 questionnaire for assessing autonomic symptoms. Moreover, patients were followed up for a sufficient period (6 months) to detect changes in these parameters as a result of an intervention [24].

However, the present study also has some limitations. First, the absence of a control group and the study design – a sub-study within the context of a larger randomised controlled trial on physical activity coaching in patients with COPD – did not allow us to explore in more detail the causal relationship between changes in physical activity and changes in cardiac autonomic function. Second, there was no strict control over parameters influencing the autonomic nervous system (i.e., medication, caffeine, circadian rhythm, sleep quality, stress and comorbidities) and although patients were questioned weekly about changes in medication, we cannot confirm that doses of short-acting beta-2 mimetics remained unchanged throughout the follow-up period. However, since the primary analysis focused on RHR and HRV measured during sleep, this may only have had a limited impact. Furthermore, we conducted additional analyses excluding patients using β-blockers as well as patients diagnosed with sleep apnoea, as both conditions are known to affect cardiac autonomic function. However, we chose not to exclude these subgroups from the main analysis in order to preserve the representativeness of the sample and strengthen the external validity of our findings. Third, most patients had mild to moderate COPD. Thus, the present results cannot be extrapolated to the COPD population and more specifically to patients with a more severe disease. Finally, due to technical issues, data from 3 patients at baseline and 7 patients at 6 months follow-up were missing. Therefore, we measured parameters of cardiac autonomic function during the clinical visit as well, including a larger sample. The resulting small sample size precludes making strong conclusions. As such, although our study was not powered to detect small effect sizes, and no formal power analysis was performed, we provided effect sizes throughout as a measure of association strength.

## Conclusion

Parameters of cardiac autonomic function are only associated to a limited extent with both the amount and intensity of physical activity, as well as exercise capacity. Exercise capacity and movement intensity were associated with resting heart rate and heart rate recovery in patients with COPD. The amount of physical activity was associated to heart rate variability. Patients who increased their amount of physical activity during 6 months of physical activity coaching showed a decrease in resting heart rate and improved autonomic balance when not on β-blockers.

## Supporting information

S1 Table Baseline characteristics of patients included in the 24 hour assessment, expressed as mean ± SD, median (Q1-Q3) or proportions. (DOCX)

S2 Table Baseline characteristics of patients who completed the study versus patients who did not, expressed as mean ± SD, median (Q1-Q3) or proportions. (DOCX)

S3 Table Changes (median (Q1-Q3)) in parameters of autonomic function in patients with COPD not taking β-blockers between baseline and 6-months follow-up (6M). (DOCX)

S4 Table Baseline characteristics of patients included in the 5 minute assessment, expressed as mean ± SD, median (Q1-Q3) or proportions. (DOCX)

S5 Table Changes (median (Q1-Q3)) in parameters of autonomic function measured during 5 minute assessment in patients with COPD between baseline and 6-months follow-up (6M). (DOCX)

S1 Fig Spearman correlations between autonomic function (24 hour assessment) and functional exercise capacity or physical activity in patients with COPD not taking β-blockers at baseline. (DOCX)

S2 Fig Spearman correlations between changes in physical activity and changes in autonomic function (24 hour assessment) in patients with COPD not taking β-blockers between baseline and 6-months follow-up. (DOCX)

S3 Fig Spearman correlations between autonomic function (5 minute assessment) and functional exercise capacity or physical activity in patients with COPD at baseline. (DOCX)

S4 Fig Spearman correlations between changes in physical activity and changes in autonomic function (5 minute assessment) in patients with COPD between baseline and 6-months follow-up. (DOCX)

## Abbreviations

6MWD: Six-minute walking distance
6MWT: Six-minute walking test
ATS: American thoracic society
bpm: Beats per minute
CAT: COPD assessment test
COMPASS 31: Composite autonomic symptom score
COPD: Chronic obstructive pulmonary disease
ERS: European Respiratory society
FEV_1_: Forced expiratory volume in the first second
FVC: Forced vital capacity
GOLD: Global initiative for chronic lung disease
HF: High frequency
HRR: Heart rate recovery
HRV: Heart rate variability
LF: Low frequency
m: Meters
MI: Movement intensity
mMRC: Modified medical research council
ms: Milliseconds
RHR: Resting heart rate
RMSSD: Root mean square of successive RR interval differences
r_s_: Spearman correlation
SD: Standard deviation
SDNN: Standard deviation of RR intervals
