# Supplementary material for: The relation between physical activity and cardiac autonomic dysfunction in patients with chronic obstructive pulmonary disease
Source: PLoS One. 2026 Aug 25;21(8):e0353482. doi: 10.1371/journal.pone.0353482 (PMC13505929; doi:10.1371/journal.pone.0353482)
Supplement: Supplementary file 1 [file pone.0353482.s001.docx]

**Table S1.** **Baseline characteristics of patients included in the 24 hour assessment**, expressed as mean ± SD, median (Q1-Q3) or proportions.

| **Variable** | **Patients with COPD**  **(n = 33)** | | |
| --- | --- | --- | --- |
| **Age (years)** | 70 | ± | 7 |
| **Male, n (%)** | 24 (73) | | |
| **BMI (kg/m^2^)** | 27 | ± | 6 |
| **FEV_1_ (% pred)** | 54 | ± | 13 |
| **GOLD stage**  **I-II, n (%)**  **III-IV, n (%)** | 20 (61)  13 (39) | | |
| **Frequent exacerbators, n (%)** | 4 (12) | | |
| **Current smokers, n (%)** | 2 (6) | | |
| **β-blockers, n (%)** | 10 (30) | | |
| **Obstructive sleep apnoea syndrome, n (%)** | 7 (21) | | |
| **6MWD (m)** | 481 | ± | 89 |
| **6MWD (%pred)** | 78 | ± | 12 |
| **Quadriceps force (%pred)** | 78 | ± | 21 |
| **COMPASS 31 (score, 0-100)** | 19 | ± | 13 |
| **mMRC (score, 0-4)** | 2 (1-3) | | |
| **CAT (score, 0-40)** | 18 | ± | 5 |
| **Mean steps per day (n/day)** | 5108 | ± | 2466 |
| **Movement intensity during walking (m/s²)** | 1.7 | ± | 0.2 |

6MWD= Six-minute walking distance; BMI= Body mass index; CAT= COPD assessment test, a higher score indicating a worser health status; COMPASS 31 questionnaire= composite autonomic symptom score, a higher score indicating more symptoms of autonomic dysfunction; FEV_1_= Forced expiratory volume in the first second; GOLD= Global initiative for chronic obstructive lung disease; mMRC= modified medical research council dyspnoea scale, a higher score indicating more dyspnoea.
