# Supplementary material for: The relation between physical activity and cardiac autonomic dysfunction in patients with chronic obstructive pulmonary disease
Source: PLoS One. 2026 Aug 25;21(8):e0353482. doi: 10.1371/journal.pone.0353482 (PMC13505929; doi:10.1371/journal.pone.0353482)
Supplement: Supplementary file 2 [file pone.0353482.s002.docx]

**Table S2.** **Baseline characteristics of patients who completed the study versus patients who did not**, expressed as mean ± SD, median (Q1-Q3) or proportions.

| **Variable** | **Patients who completed**  **(n = 26)** | | | **Patients who not completed (n = 10)** | | | **p-value** |
| --- | --- | --- | --- | --- | --- | --- | --- |
| **Age (years)** | 68 | ± | 6 | 71 | ± | 10 | 0.46 |
| **Male, n (%)** | 19 (73) | | | 6 (60) | | |  |
| **BMI (kg/m^2^)** | 27 | ± | 6 | 28 | ± | 6 | 0.81 |
| **FEV_1_ (% pred)** | 56 | ± | 13 | 46 | ± | 12 | 0.04* |
| **GOLD stage**  **I-II, n (%)**  **III-IV, n (%)** | 16 (62)  10 (38) | | | 8 (80)  2 (20) | | | 0.10 |
| **Frequent exacerbators, n (%)** | 2 (8) | | | 2 (20) | | |  |
| **Current smokers, n (%)** | 2 (8) | | | 1 (10) | | |  |
| **6MWD (m)** | 499 | ± | 73 | 420 | ± | 102 | 0.01* |
| **6MWD (%pred)** | 80 | ± | 10 | 70 | ± | 13 | 0.03* |
| **Quadriceps force (%pred)** | 81 | ± | 18 | 69 | ± | 23 | 0.10 |
| **COMPASS 31 (score, 0-100)** | 19 | ± | 13 | 18 | ± | 13 | 0.95 |
| **mMRC (score, 0-4)** | 2 (1-2) | | | 2 (1-3) | | | 0.31 |
| **CAT (score, 0-40)** | 17 | ± | 5 | 18 | ± | 5 | 0.86 |
| **Mean steps per day (n/day)** | 5280 | ± | 2630 | 4640 | ± | 2348 | 0.52 |
| **Movement intensity during walking (m/s²)** | 1.8 | ± | 0.2 | 1.6 | ± | 0.2 | 0.09 |
| **Resting heart rate (bpm)** | 67 (63-73) | | | 68 (63-76) | | | 0.34 |
| **Heart rate variability** |  |  |  |  |  |  |  |
| **SDNN (ms)** | 24 (15-36) | | | 26 (22-27) | | | 0.43 |
| **RMSSD (ms)** | 14 (12-33) | | | 26 (16-27) | | | 0.34 |
| **LF/HF ratio** | 2 (1-6) | | | 1 (1-4) | | | 0.14 |
| **SD2/SD1 ratio** | 2 (2-3) | | | 2 (2-3) | | | 0.20 |
| **Heart rate recovery (bpm)** | 14 (10-19) | | | 11 (6-16) | | | 0.14 |

6MWD= Six-minute walking distance; BMI= Body mass index; bpm= Beats per minute; CAT= COPD assessment test, a higher score indicating a worser health status; COMPASS 31 questionnaire= composite autonomic symptom score, a higher score indicating more symptoms of autonomic dysfunction; FEV_1_= Forced expiratory volume in the first second; GOLD= Global initiative for chronic obstructive lung disease; LF/HF= Low frequency/High frequency; mMRC= modified medical research council dyspnoea scale, a higher score indicating more dyspnoea; ms= Milliseconds; RMSSD =Root mean square of successive RR interval differences; SD1= Short-term variability (standard deviation perpendicular to the line of identity); SD2= Long-term variability (standard deviation along the line of identity); SDNN= Standard deviation of RR intervals. *= Statistically significant (p-value < 0.05).
