# Supplementary material for: The relation between physical activity and cardiac autonomic dysfunction in patients with chronic obstructive pulmonary disease
Source: PLoS One. 2026 Aug 25;21(8):e0353482. doi: 10.1371/journal.pone.0353482 (PMC13505929; doi:10.1371/journal.pone.0353482)
Supplement: Supplementary file 3 [file pone.0353482.s003.docx]

**Table S3.** **Changes (median (Q1-Q3)) in parameters of autonomic function in patients with COPD not taking β-blockers between baseline and 6-months follow-up (6M).**

| **Parameter** | **Baseline**  **Median (Q1-Q3)** | **6M**  **Median (Q1-Q3)** | **∆**  **Median (Q1-Q3)** | **p-value** |
| --- | --- | --- | --- | --- |
| 6MWD (m) | 520 (450-561) | 510 (433-545) | -11 (-24-12) | 0.27 |
| Mean steps per day (n/day) | 4948 (3300-7216) | 5071 (3059-7571) | 583 (-632-1324) | 0.20 |
| MI during walking (m/s²) | 1.73 (1.54-1.87) | 1.65 (1.51-1.97) | 0.00 (-0.09-0.09) | 0.94 |
| Heart rate variability |  |  |  |  |
| SDNN (ms) | 23 (18-34) | 25 (21-34) | 0 (-1-6) | 0.55 |
| RMSSD (ms) | 14 (12-31) | 26 (16-29) | 1 (-2-6) | 0.31 |
| LF/HF ratio | 2 (1-6) | 3 (1-5) | 0 (-1-1) | 0.78 |
| SD2/SD1 ratio | 2 (2-3) | 2 (1-3) | 0 (0-0) | 0.46 |
| Heart rate recovery (bpm) | 14 (11-19) | 14 (8-21) | 1 (-5-5) | 0.99 |
| COMPASS 31 |  |  |  |  |
| Total score | 13 (8-27) | 17 (6-23) | -1 (-5-3) | 0.35 |
| Orthostatic function | 0 (0-16) | 0 (0-12) | 0 (0-4) | 0.78 |
| Vasomotor function | 0 (0-0) | 0 (0-2) | 0 (0-0) | 0.99 |
| Secretomotor function | 4 (0-6) | 2 (0-6) | 0 (-2-0) | 0.63 |
| Gastrointestinal function | 3 (1-6) | 2 (0-5) | 0 (-2-1) | 0.25 |
| Bladder function | 1 (0-2) | 0 (0-2) | 0 (-1-0) | 0.05* |
| Pupillomotor function | 1 (1-2) | 1 (1-2) | 0 (-1-1) | 0.99 |

6MWD= Six-minute walking distance; bpm= Beats per minute; COMPASS 31 questionnaire (0-100), a higher score indicating more symptoms of autonomic dysfunction; Orthostatic function (0-40); Vasomotor function (0-5); Secretomotor function (0-15); Gastrointestinal function (0-25); Bladder function (0-10) and Pupillomotor function (0-5); LF/HF= Low frequency/High frequency; MI= Movement intensity; ms= Milliseconds; RMSSD =Root mean square of successive RR interval differences; SD1= Short-term variability (standard deviation perpendicular to the line of identity); SD2= Long-term variability (standard deviation along the line of identity); SDNN= Standard deviation of RR intervals. *= Statistically significant (p-value < 0.05).
