# Supplementary material for: The relation between physical activity and cardiac autonomic dysfunction in patients with chronic obstructive pulmonary disease
Source: PLoS One. 2026 Aug 25;21(8):e0353482. doi: 10.1371/journal.pone.0353482 (PMC13505929; doi:10.1371/journal.pone.0353482)
Supplement: Supplementary file 5 [file pone.0353482.s005.docx]

**Table S5.** **Changes (median (Q1-Q3)) in parameters of autonomic function measured during 5 minute assessment in patients with COPD between baseline and 6-months follow-up (6M).**

| **Parameter** | **Baseline**  **Median (Q1-Q3)** | **6M**  **Median (Q1-Q3)** | **∆**  **Median (Q1-Q3)** | **p-value** |
| --- | --- | --- | --- | --- |
| 6MWD (m) | 512 (434-545) | 495 (436-539) | 4 (-17-22) | 0.92 |
| Mean steps/day | 5152 (3300-6996) | 5004 (3354-7571) | 550 (-632-1324) | 0.19 |
| MI during walking (m/s²) | 1.71 (1.54-1.87) | 1.63 (1.50-1.97) | 0 (-0.09-0.09) | 0.80 |
| Resting heart rate |  |  |  |  |
| No β-blocker use (bpm) | 71 (67-78) | 71 (65-84) | -2 (-4-7) | 0.81 |
| β-blocker use (bpm) | 63 (58-77) | 62 (60-65) | -6 (-8-2) | 0.20 |
| Heart rate variability |  |  |  |  |
| SDNN (ms) | 21 (15-35) | 18 (11-28) | -3 (-9-1) | 0.01* |
| RMSSD (ms) | 21 (11-38) | 14 (11-23) | -3 (-12-0) | 0.01* |
| LF/HF ratio | 1 (0-4) | 1 (1-5) | 0 (-2-1) | 0.23 |
| SD2/SD1 ratio | 2 (1-2) | 2 (1-3) | 0 (-1-1) | 0.58 |

Bpm= Beats per minute; COMPASS 31 questionnaire (0-100), a higher score indicating more symptoms of autonomic dysfunction; Orthostatic function (0-40); Vasomotor function (0-5); Secretomotor function (0-15); Gastrointestinal function (0-25); Bladder function (0-10) and Pupillomotor function (0-5); LF/HF= Low frequency/High frequency; ms= Milliseconds; RMSSD =Root mean square of successive RR interval differences; SD1= Short-term variability (standard deviation perpendicular to the line of identity); SD2= Long-term variability (standard deviation along the line of identity); SDNN= Standard deviation of RR intervals. *= Statistically significant (p-value < 0.05).
