# Supplementary material for: The relation between physical activity and cardiac autonomic dysfunction in patients with chronic obstructive pulmonary disease
Source: PLoS One. 2026 Aug 25;21(8):e0353482. doi: 10.1371/journal.pone.0353482 (PMC13505929; doi:10.1371/journal.pone.0353482)
Supplement: Supplementary file 6 [file pone.0353482.s006.docx]

**Fig S1.** **Spearman correlations between autonomic function (24 hour assessment) and functional exercise capacity or physical activity in patients with COPD not taking β-blockers at baseline.** Red= negative correlation and blue= positive correlation, with a darker colour representing a stronger correlation. 6MWD= Six-minute walking distance; bpm= Beats per minute; HRV= Heart rate variability; LF/HF= Low frequency/High frequency; MI= Movement intensity; ms= Milliseconds; RHR= Resting heart rate; RMSSD=Root mean square of successive RR interval differences; SD1= Short-term variability (standard deviation perpendicular to the line of identity); SD2= Long-term variability (standard deviation along the line of identity); SDNN= Standard deviation of RR intervals.
