# Supplementary material for: The relation between physical activity and cardiac autonomic dysfunction in patients with chronic obstructive pulmonary disease
Source: PLoS One. 2026 Aug 25;21(8):e0353482. doi: 10.1371/journal.pone.0353482 (PMC13505929; doi:10.1371/journal.pone.0353482)
Supplement: Supplementary file 7 [file pone.0353482.s007.docx]

**Fig S2. Spearman correlations between changes in physical activity and changes in autonomic function (24 hour assessment) in patients with COPD not taking β-blockers between baseline and 6-months follow-up.** Red= negative correlation and blue= positive correlation, with a darker colour representing a stronger correlation. bpm= Beats per minute; HRV= Heart rate variability; LF/HF= Low frequency/High frequency; MI= Movement intensity; ms= Milliseconds; RHR= Resting heart rate; RMSSD=Root mean square of successive RR interval differences; SD1= Short-term variability (standard deviation perpendicular to the line of identity); SD2= Long-term variability (standard deviation along the line of identity); SDNN= Standard deviation of RR intervals. *= Statistically significant (p-value < 0.05); ^§^= Trend towards significance (p-value < 0.10).
