# Supplementary material for: The relation between physical activity and cardiac autonomic dysfunction in patients with chronic obstructive pulmonary disease
Source: PLoS One. 2026 Aug 25;21(8):e0353482. doi: 10.1371/journal.pone.0353482 (PMC13505929; doi:10.1371/journal.pone.0353482)
Supplement: Supplementary file 8 [file pone.0353482.s008.docx]

**Fig S3. Spearman correlations between autonomic function (5 minute assessment) and functional exercise capacity or physical activity in patients with COPD at baseline.** Red= negative correlation and blue= positive correlation, with a darker colour representing a stronger correlation. 6MWD= Six-minute walking distance; bpm= Beats per minute; HRV= Heart rate variability; LF/HF= Low frequency/High frequency; MI= Movement intensity; ms= Milliseconds; RHR= Resting heart rate; RMSSD=Root mean square of successive RR interval differences; SD1= Short-term variability (standard deviation perpendicular to the line of identity); SD2= Long-term variability (standard deviation along the line of identity); SDNN= Standard deviation of RR intervals. *= Statistically significant (p-value < 0.05); ^§^= Trend towards significance (p-value < 0.10).

The sensitivity analysis showed that MI during walking was weakly associated with RHR (r_s_=-0.37) (Figure S3). This association remained when excluding patients taking β-blockers (r_s_=-0.37). Additionally, a weak association between MI during walking and SD2/SD1 ratio was found (r_s_=0.31). Excluding patients on stable doses of β-blockers showed weak associations between the functional exercise capacity and RHR (r_s_=-0.36) as well as HRV, expressed as SDNN and LF/HF-ratio (r_s_=0.35 and r_s_=0.30, respectively). The amount of physical activity was weakly associated with HRV, expressed as LF/HF ratio (r_s_=0.30).
