# Supplementary material for: The relation between physical activity and cardiac autonomic dysfunction in patients with chronic obstructive pulmonary disease
Source: PLoS One. 2026 Aug 25;21(8):e0353482. doi: 10.1371/journal.pone.0353482 (PMC13505929; doi:10.1371/journal.pone.0353482)
Supplement: Supplementary file 9 [file pone.0353482.s009.docx]

**Fig S4.** **Spearman correlations between changes in physical activity and changes in autonomic function (5 minute assessment) in patients with COPD between baseline and 6-months follow-up.** Red= negative correlation and blue= positive correlation, with a darker colour representing a stronger correlation. bpm= Beats per minute; HRV= Heart rate variability; LF/HF= Low frequency/High frequency; MI= Movement intensity; ms= Milliseconds; RHR= Resting heart rate; RMSSD=Root mean square of successive RR interval differences; SD1= Short-term variability (standard deviation perpendicular to the line of identity); SD2= Long-term variability (standard deviation along the line of identity); SDNN= Standard deviation of RR intervals. ^§^= Trend towards significance (p-value < 0.10).

SDNN and RMSSD significantly decreased between baseline and 6 months follow-up (∆ -3 (-9-1) milliseconds, p=0.010 and ∆ -3 (-12-0) milliseconds, p=0.006, respectively) (Table S4). Changes in physical activity were not related to changes in HRV. The change in mean steps per day was inversely associated with the change in RHR including the total population (r_s_=-0.34) (Figure S4) as well as when excluding patients on stable doses of β-blockers (r_s_=-0.51).
